# Study on Springback Behavior in Hydroforming of Micro Channels for a Metal Bipolar Plate

**DOI:** 10.3390/ma17215386

**Published:** 2024-11-04

**Authors:** Zonghui Su, Wenlong Xie, Yong Xu, Changsheng Li, Liangliang Xia, Baocheng Yang, Mingyu Gao, Hongwu Song, Shihong Zhang

**Affiliations:** 1 State Key Laboratory of Rolling and Automation, Northeastern University, Shenyang 110819, China; 17855599285@163.com (Z.S.); lics@ral.neu.edu.cn (C.L.); 2 Institute of Metal Research, Chinese Academy of Sciences, Shenyang 110016, China; wlxie17b@imr.ac.cn (W.X.); bchyang19b@imr.ac.cn (B.Y.); mygao21s@imr.ac.cn (M.G.); hwsong@imr.ac.cn (H.S.); shzhang@imr.ac.cn (S.Z.); 3 Shenyang Key Laboratory for Aerospace Complex Components Precision Manufacturing, Shenyang 110016, China; 4 College of Transportation, Ludong University, Yantai 264025, China; llxia17b@imr.ac.cn

**Keywords:** bipolar plate, springback, hydroforming, deformation degree, grain size

## Abstract

Bipolar plates are one of the most important components of proton exchange membrane fuel cells. With the miniaturization of bipolar plate flow channel sizes and the increasing demand for precision, springback has become a key focus of research in the bipolar plate forming process. In this paper, the hydroforming process for 316L stainless steel bipolar plates was studied, and an FEM model was built to examine the stress and strain at various locations on the longitudinal section of the plate. Modeling accuracy was validated by the comparison of experimental profile and thickness distribution. The effects of forming pressure and grain size on springback behavior are discussed. The results show that with increasing forming pressure, the springback value decreases initially, followed by an increase, but then again decreases. When the forming pressure is 80 MPa–100 MPa, the deformation of the lower element of the upper rounded corner is not uniform with more elastic regions, and the springback is positively correlated with forming pressure. The springback distribution pattern on the cross-section of the bipolar plate changes from a normal distribution to a distribution of “M” shape with increased pressure. The larger the grain size, the lower the yield strength elastic proportion, resulting in a decrease in springback of the sheet. The maximum amount of springback of the bipolar plate is 3.1 μm when the grain size is 60.7 μm. The research results provide a reference for improving the forming quality of metal bipolar plates with different flow channel shapes.

## 1. Introduction

The development and utilization of hydrogen energy is an important approach to address the energy crisis [1,2]. The hydrogen-oxygen fuel cell is a typical way to utilize hydrogen energy. It has the advantages of high energy efficiency, high energy conversion rate, and low pollution [3]. The proton exchange membrane fuel cell (PEMFC) is a typical representative of hydrogen energy fuel cells in which the bipolar plates are the core components. The main functions of the bipolar plates [4,5,6] include providing flow channels for hydrogen and oxygen, separating the two gases, and collecting electrons. As a result, bipolar plates need to have good air tightness, good electrical and thermal conductivity, low resistance, good chemical stability and high corrosion resistance while meeting the requirements of low production cost and high-volume production [7,8]. Metal bipolar plates have been attracting attention and are considered ideal for the industrialization of PEMFC due to their good electrical and thermal conductivity and high strength [9,10].

Forming processes for metal bipolar plates mainly include stamping, rubber forming, roll forming, and hydroforming [11]. Stamping [12,13,14,15,16] is the most common method of forming bipolar plates. Hu et al. [14] used finite element software to analyze the thickness changes during the stamping process and predicted potential defects in the forming process. Lan et al. [15] studied the influences of the various factors of die sets on the forming behavior of the wave-like channel and proposed methods to improve the forming quality. Zhu et al. [16] proposed a two-step stamping method for the fabrication of titanium bipolar plates, which led to improvements in ultimate depth, thickness uniformity and dimensional accuracy.

Rubber forming [17,18,19,20] is similar to stamping, except that the rigid die in stamping is replaced with elastic material. Peng et al. [21] conducted a detailed study on the influence of grain size, lubrication conditions, and punch hardness on the forming quality of sheet metal during the soft punch forming process. Liu Y et al. [22] utilized the finite element model to investigate the impact of major parameters such as rubber hardness and key geometric dimensions of the rigid die on the forming results.

Roll forming [23,24] utilizes the point-to-point conjugate principle to engrave channels on the rolls. Through the force between the two rolls, channels are formed on the surface of the sheet metal. This forming method is highly efficient but the sheet material is prone to warping deformation during the roll forming process, which seriously affects the forming quality of the bipolar plate. Moreover, this method is not suitable for the manufacture of complex flow channels [25].

Hydroforming is another method of manufacturing bipolar plates. Mohammadtabar et al. [26] proposed two-step hydroforming for fabricating bipolar plates and showed that the forming depth and filling percentage could be significantly increased. Xu et al. [27] investigated the effect of grain size on the forming results of pure copper bipolar plates. Zhang et al. [28] found that the forming effect of the outer radius of the die was superior to that of the inner radius, and the forming effect on the bipolar plate can be improved by increasing the outer radius. Wang Y. et al. [29] proposed a new method for ultra-thin TA1/CFRP laminate low-constraint hydro-microforming. The results show that 40 °C is the optimum forming temperature, and wet friction conditions are more favorable to forming quality than dry friction conditions. Belali et al. [30] investigated the forming of sheet metal with pin patterns by hydroforming, stamping, and hydroforming-stamping methods. The results of the study showed that hydroforming-stamping gave the best forming results.

Microforming of ultra-thin metal bipolar plates is a complicated problem. Various defects may occur during sheet metal forming, including fracture [31,32], wrinkling [33] and springback [34,35,36,37]. Numerous scholars have carried out a lot of theoretical research and experimental exploration from the perspective of forming mechanisms and process optimization. With the miniaturization of bipolar plate flow channel sizes and the increasing demand for precision, springback has become a key focus of research in the bipolar plate forming process. This paper takes 316L stainless steel sheet of thickness 0.075 mm as the research object. The influence of deformation degree and grain size on the forming and springback behavior of the bipolar plate during the hydroforming process was investigated. The results of this paper can provide a reference for the hydroforming preparation and engineering application of ultra-thin metal bipolar plates.

## 2. Materials and Methods

### 2.1. Research Objectives

**Figure 1:**
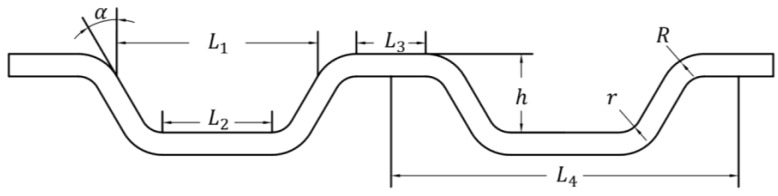
Geometry of bipolar plate channel.

The size parameters of the bipolar plate microchannel studied in this paper are shown in Figure 1, which include the channel width ($L_{1}$), bottom width *(*$L_{2}$), channel ridge width *(*$L_{3}$), channel depth (*h*), upper fillet radius (*R*), bottom fillet radius (*r)*, draft angle (*α*) and term ($L_{4}$). The values of size parameters are shown in Table 1.

### 2.2. Experimental Materials

In this paper, cold-rolled annealed 316L stainless steel with a thickness of 0.075 mm is used. Its chemical composition was determined by utilizing an iCAP6300 Inductively Coupled Plasma Atomic Emission Spectrometer (Manufactured by Thermo Fisher Scientific, Waltham, MA, USA) and CS600 Carbon sulfur analyzer (Manufactured by Laboratory Equipment Corporation, St. Joseph, MI, USA), and the results are shown in Table 2.

**Figure 2:**
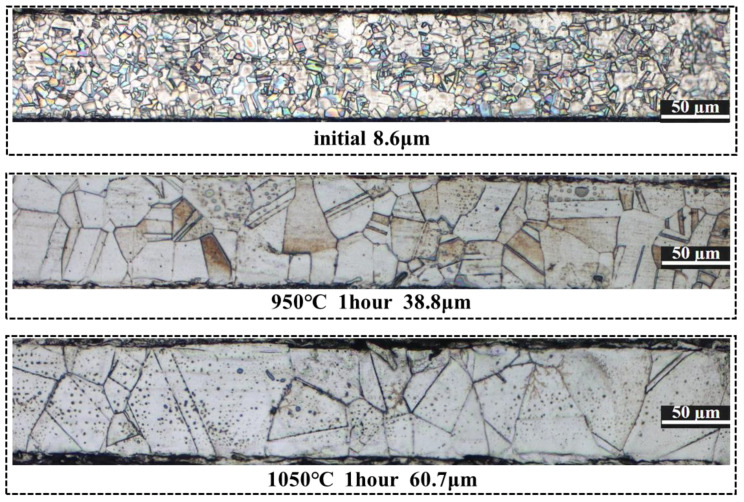
Metallographic pictures of 316L stainless steel in different states.

The heat treatment of the materials used in this study was carried out at 950 °C for 1 h with water cooling, or 1050 °C for 1 h with water cooling. The metallographic pictures of the initial state and different heat treatment states of 316L stainless steel are shown in Figure 2. As measured, the grain sizes of the initial state plates, 950 °C heat-treated, and 1050 °C heat-treated plates were 8.6 μm, 38.8 μm and 60.7 μm, respectively.

**Figure 3:**
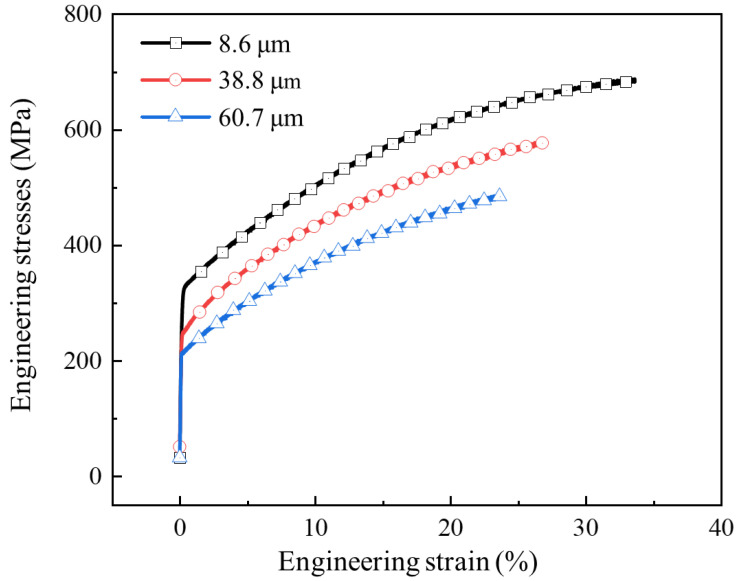
Engineering stress-strain curve of 316L stainless steel with different grain sizes.

The mechanical properties of 316L stainless steel were obtained through uniaxial tensile tests with a strain rate of 10^−3^s^−1^, and the results are presented in Figure 3. The stress-strain relationship under different grain sizes is described by Equation (1), and the mechanical property parameters of the sheets with different grain sizes are shown in Table 3.

(1) $$\sigma =K{\left(\varepsilon_{0}+{\bar{\varepsilon }}^{p}\right)}^{n}$$

where *K* is the strength coefficient, n is the strain-hardening exponent, and $\varepsilon_{0}$ is the initial strain.

### 2.3. Experimental Equipment

**Figure 4:**
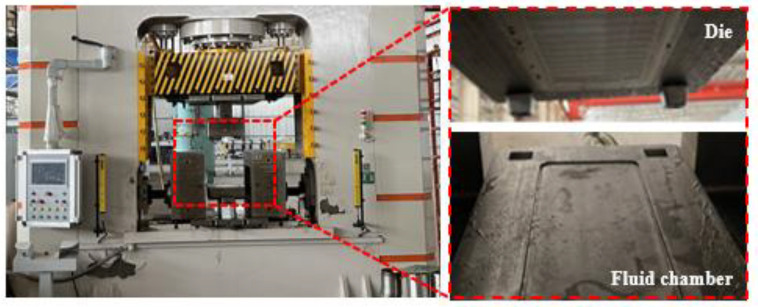
Experimental setup for hydroforming of 316L stainless steel bipolar plates.

The experiments were conducted on the 1000 t hydroforming machine, and the experimental setup is shown in Figure 4. The die is located above the interior of the equipment and the fluid chamber is located below the interior. During the experiment, the sheet is placed on the top of the fluid chamber, and the die moves downward to complete the closing process. High-pressure fluid in the fluid chamber drives the sheet to fit the die and complete the bipolar plate forming process. During the process, the filling parameters can be controlled through the operation console to vary the experimental conditions.

### 2.4. Finite Element Model

**Figure 5:**
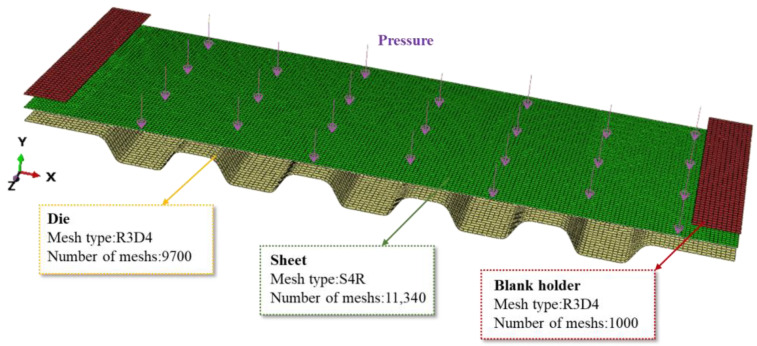
Finite element model for hydroforming of bipolar plate.

The bipolar plate has many flow paths in the actual forming process; five channels with typical features are selected for modeling in the finite element simulation in order to save computational time. Blank holders are applied to increase the material constraints during the molding process, since the material in the middle runner position is also constrained during the actual forming. The established three-dimensional finite element model for hydroforming of the bipolar plate is shown in Figure 5. The die was constructed according to the channel parameters in Table 1. The friction coefficient between the blank holds and the sheet, as well as between the die and the sheet, was set at 0.1. A uniform load was applied to the sheet to simulate the high-pressure fluid in the fluid chamber. The type and number of mesh elements in the finite element model is shown in Figure 5.

### 2.5. Finite Element Model Accuracy Verification

The accuracy of the finite element model was verified in terms of the profile of the formed channel and the thickness distribution of the formed channel after forming. The finite element model and the experimental setup were set to the same experimental parameters; non-heat-treated sheet metal was selected, and the liquid chamber pressure was set to 100 MPa.

**Figure 6:**
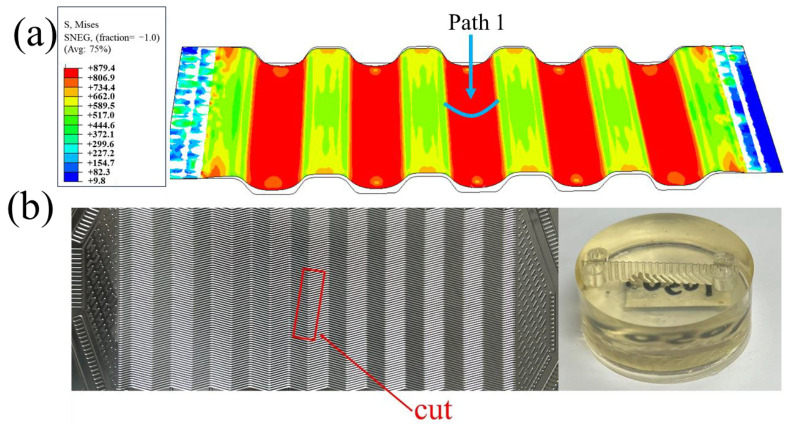
(**a**) Simulation results. (**b**) Schematic of the sample.

**Figure 7:**
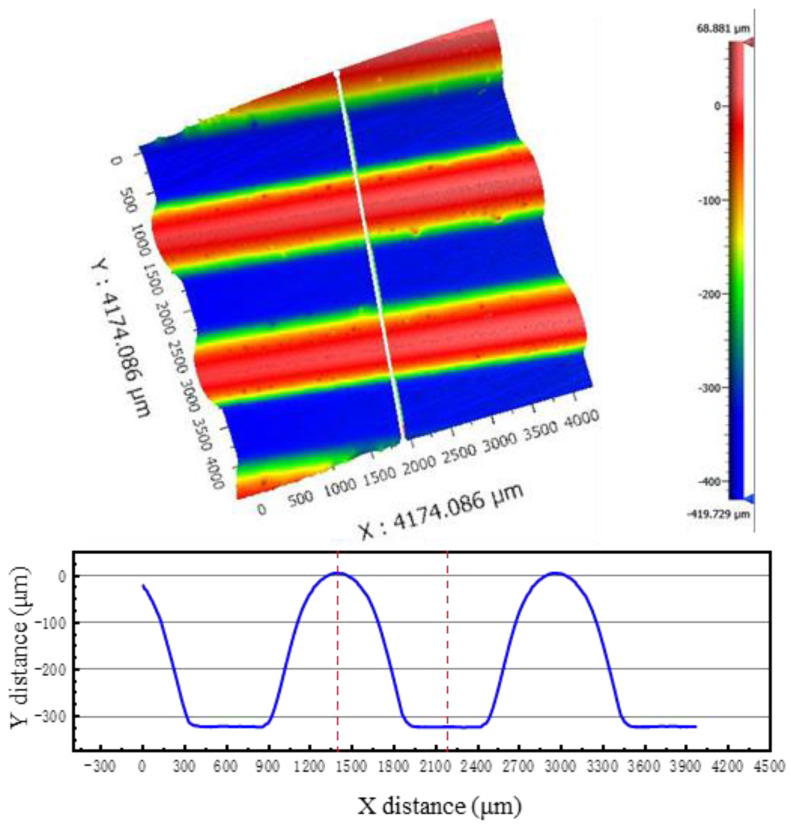
White light interferometry results of channel profile.

**Figure 8:**
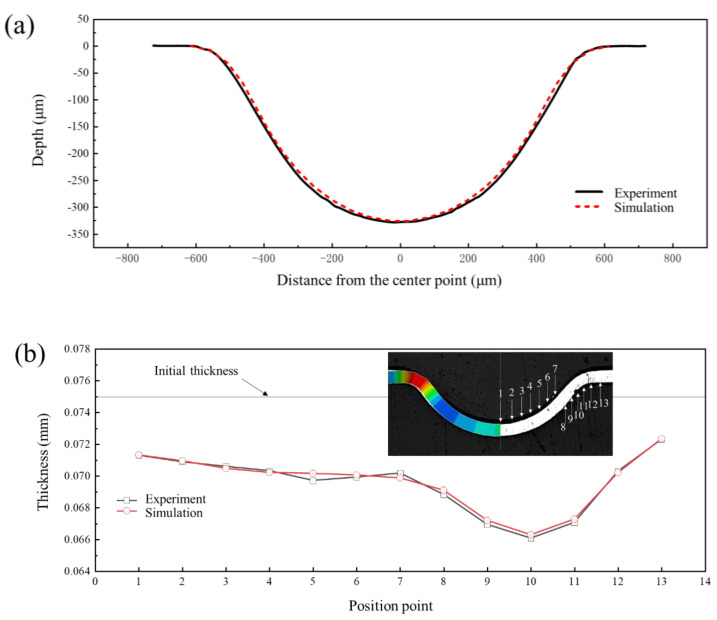
Comparison of experiment and simulation results: (**a**) profile distribution; (**b**) thickness distribution.

In order to ensure the stability of simulation results during comparison, “Path 1” was generated at the middle position of the middle channel in Figure 6a. The profile distribution and the thickness distribution on this path were extracted. White light interferometry was used to measure the formed channel profile in the experiment to compare the accuracy of the depth and shape of the channel profile (Figure 7). In order to compare the accuracy of the simulation results in terms of thickness, the formed bipolar plate was cut into 10 mm × 20 mm samples along the vertical flow channel by Electrical Discharge Machining, and then embedded into small samples by cold inlay, as shown in Figure 6b. The surface was polished with sandpaper until the surface was free of scratches. The formed channel morphology was observed using an Axio ObserverZ1 optical microscope, as shown in Figure 8b, and the thickness of the formed channel was measured using Image-Pro Plus 6.0 software.

The depth of the formed channel was measured using a NewView 9000 white light interferometer (Manufactured by Kla Corporation, Milpitas, CA, USA. resolution not less than 0.1 nm) and the results are shown in Figure 7. It can be seen that the forming height of the channel is 328 μm, and the profile quality is good. Figure 8 shows the experimental and simulation comparison results when the liquid pressure is 100 MPa. It is worth noting that the results in Figure 8 were obtained after springback simulation. As can be seen from the results, the depth of the channel in the experimental sample was 328 μm, while depth of the channel in the simulation was 326 μm with an error of 0.61%. In addition, there is no significant difference between the simulated and experimental shapes of the channel. Both the maximum and minimum thickness errors of the thickness results are less than 5%, and the thickness distribution is relatively consistent. Therefore, the finite element model established in this paper is accurate.

## 3. Results

### 3.1. Springback Behaviors Under Different Degrees of Deformation

**Figure 9:**
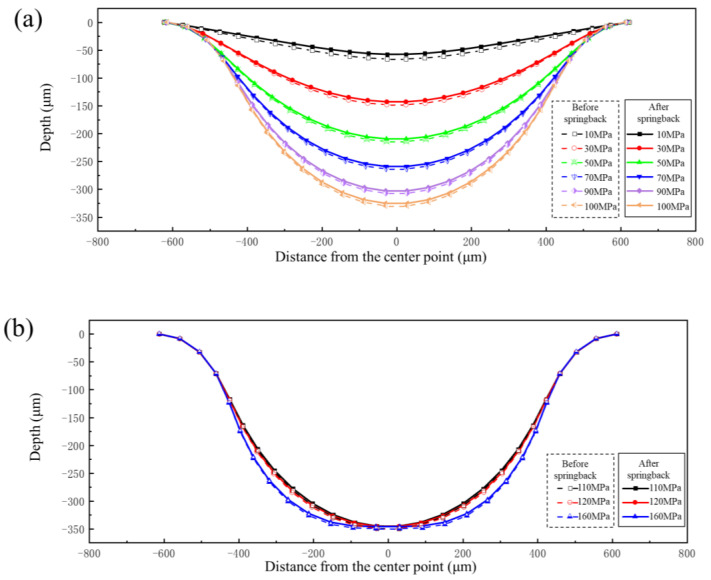
Forming profile and springback profile of bipolar plate under different internal pressures: (**a**) 10–100 MPa; (**b**) 110–160 MPa.

**Figure 10:**
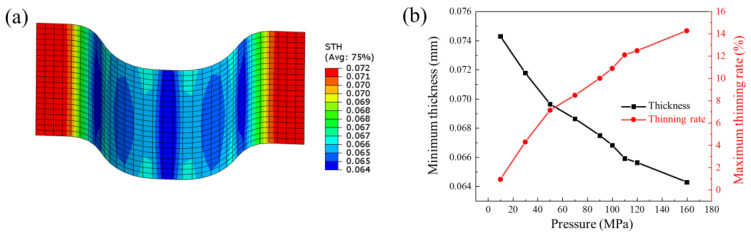
(**a**) Thickness distribution of the bipolar plate under pressure of 160 MPa; (**b**) the minimum thickness and maximum thinning rate at different forming pressures.

In the hydroforming process of bipolar plates, the forming pressure is one of the most important factors affecting forming quality. The forming pressure is the driving force behind the deformation of the sheet. Specifically, the higher the forming pressure, the greater the degree of deformation in the sheet. In order to investigate the effect of the degree of deformation on springback, forming pressures of 10 MPa, 30 MPa, 50 MPa, 70 MPa, 90 MPa, 100 MPa, 110 MPa, 120 MPa, and 160 MPa were applied to obtain different degrees of deformation. The springback values were calculated after the forming process. The forming profile and springback profile of bipolar plates under different pressures are shown in Figure 9. It can be seen from the results that the higher the forming pressure, the deeper the channel depth. The pressure at which the bottom of the sheet starts to contact the die should be greater than 100 MPa. The thickness distribution of a typical channel for a bipolar plate under forming pressure of 160 MPa is shown in Figure 10a, and the minimum thickness and maximum thinning rate at different forming pressures are shown in Figure 10b. The point of minimum thickness is located at the upper fillet. The maximum thinning rate increases accordingly with increasing degree of deformation, but the increase rate gradually slows down. In the early stages of deformation, the plate at the upper fillet is not fully attached to the die and is therefore less constrained by the die. So, the sheet at the upper fillet is mainly subjected to tensile action, resulting in a faster reduction in thickness. However, in the later stage of deformation, the sheet at the upper fillet basically fits the die. The limiting effect of the upper fillet of the die and friction lead to a gradual deceleration in the increase of the maximum thinning rate.

**Figure 11:**
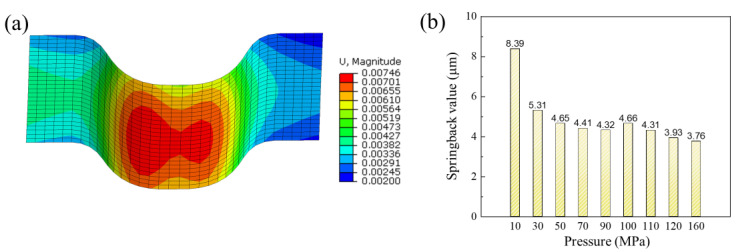
(**a**) The distribution of springback distance of a channel under pressure of 160 MPa; (**b**) the maximum springback value under different pressures.

Figure 11a shows the results of springback distance of the channel after forming under pressure of 160 MPa. The maximum springback value under different pressures is shown in Figure 11b. The results indicate that when the pressure is less than 90 MPa, the springback value decreases as the pressure increases. In the pressure range of 90 MPa to 100 MPa, the springback value shows an increasing trend with the increase in pressure. When the pressure exceeds 100 MPa, the springback value decreases as the pressure increases.

**Figure 12:**
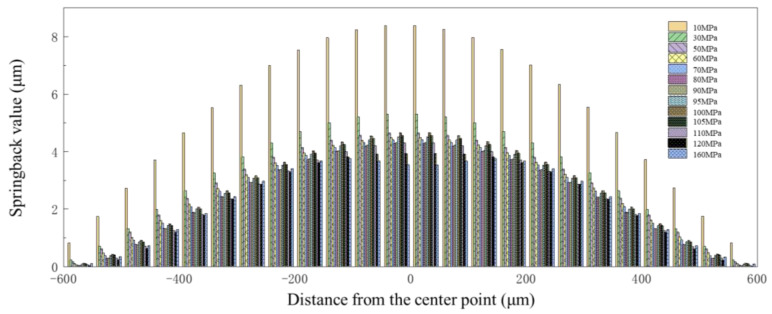
Springback value distribution along the channel under different pressures.

To ensure the reliability of the trend where the springback value decreases initially, followed by an increase, but then again decreases as the deformation degree increases, the results of springback at 60 MPa, 80 MPa, 95 MPa, and 105 MPa were obtained. The springback value was calculated at different positions along the cross-section of the channel, which is shown in Figure 12. The results clearly indicate that the changes in springback value with increasing degrees of deformation can be divided into three cases: Case 1, when the pressure is in the range of 0–80 MPa, and the springback value is negatively correlated with the degree of deformation; Case 2, when the pressure is in the range of 80–100 MPa, and the springback value is positively correlated with the degree of deformation; and Case 3, when the pressure exceeds 100 MPa, and the springback value is negatively correlated with the degree of deformation.

**Figure 13:**
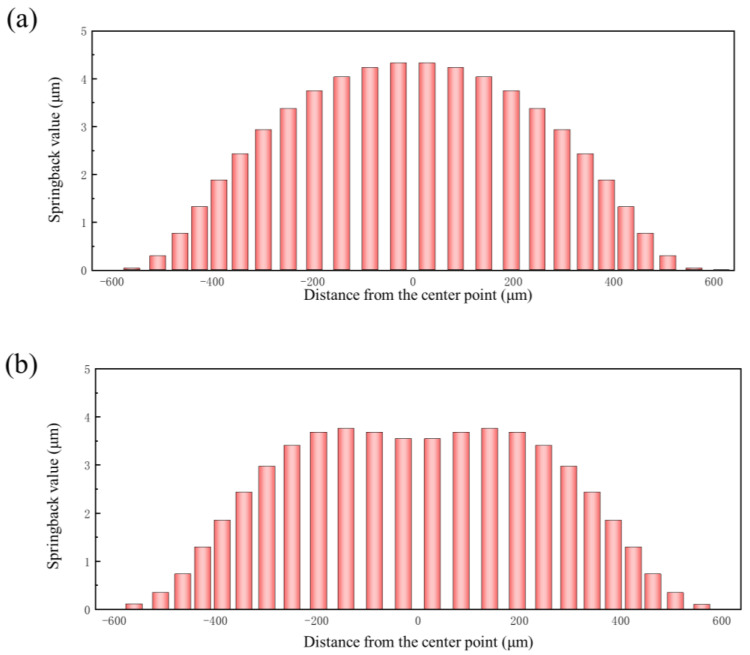
Springback value distribution along the cross-section of the channel: (**a**) 90 MPa; (**b**) 160 MPa.

The location of maximum springback is different depending on whether the bottom of the bipolar plate is attached to the die or not. As shown in Figure 13, when the deformation of the bipolar plate is not enough for the bottom of the bipolar plate to attach to the die (pressure < 100 MPa), the springback value gradually decreases from the center of the bottom towards the sides (Figure 13a). Conversely, when the deformation of the bipolar plate is enough for the bottom of the bipolar plate to attach to the die (pressure > 100 MPa), the maximum springback occurs at the two bottom fillets, and the springback value distribution along the cross-section is an “M”-shape (Figure 13b).

### 3.2. Springback Analysis Under Different Degrees of Deformation

**Figure 14:**
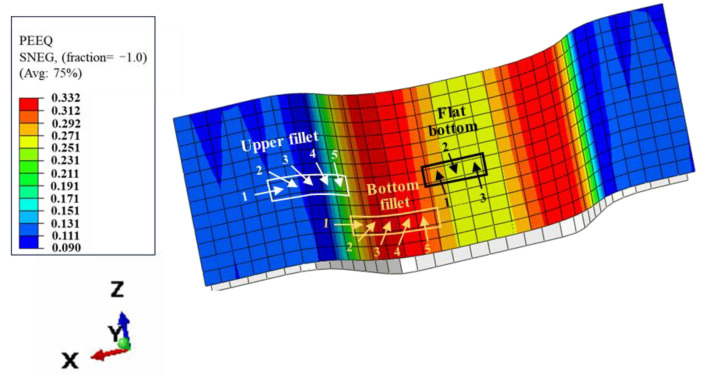
Schematic diagram of elements in different positions.

Equivalent plastic strain is used to represent the cumulative amount of plastic deformation during the forming process which will not cause springback during unloading. The elastic strain energy is recoverable strain energy, and the elastic strain energy is stored in the sheet during deformation. On unloading, the elastic strain energy is released and springback occurs. Therefore, the equivalent plastic strain and elastic strain energy of the elements on the upper fillet, bottom fillet and flat bottom of the channel were obtained to analyze the springback behavior. As shown in Figure 14, five elements on the upper fillet, five elements on the bottom fillet and three elements on the flat bottom of the channel were selected as the research objects along the cross-sectional direction.

**Figure 15:**
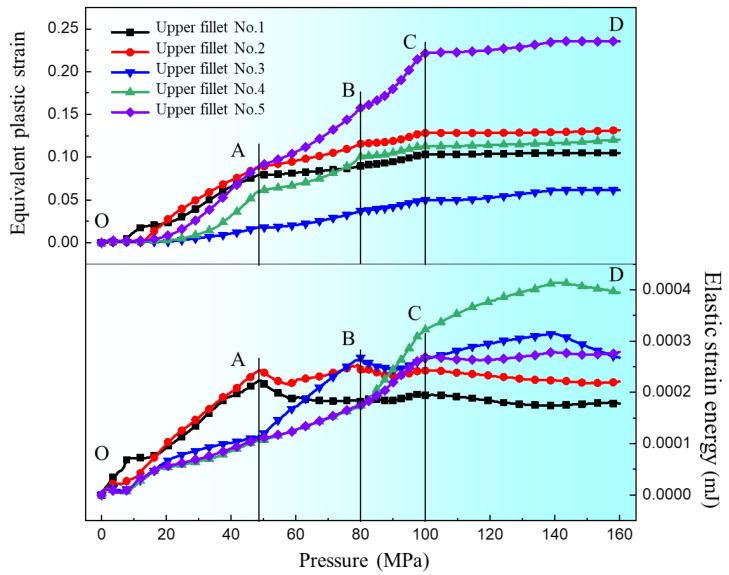
Equivalent plastic strain and elastic strain energy change with internal pressure at different points of the upper fillet.

The changes of equivalent plastic strain and elastic strain energy at different positions with increasing degrees of deformation (increase of pressure) were obtained. Figure 15 shows the equivalent plastic strain and elastic strain energy results of five elements of the upper fillet. It can be seen from the results that the equivalent plastic strain of elements 1, 2 and 5 of the upper fillet increases noticeably with the increase of pressure in the “OA” segment, and the elastic strain energy of elements 1 and 2 also increases noticeably. In the “AB” segment, the equivalent plastic strain of elements 1 and 2 of the upper fillet increases with increasing pressure, but the increase velocity slows down noticeably. However, the equivalent plastic strain of element 5 increases noticeably and the elastic strain energy value is the smallest. In this segment, the equivalent plastic strain increases while the elastic ratio decreases with increasing pressure, so the springback value decreases after unloading. This corresponds to Case 1 in Section 3.1, that is, the amount of springback is negatively correlated with the degree of deformation. In the “BC” segment, the equivalent plastic strain and elastic strain energy of elements 1, 2 and 3 do not noticeably increase with increasing degrees of deformation. At this stage, elements 1, 2 and 3 have been attached to the die and are constrained by internal pressure and friction from the die, so the deformation has basically ended. The equivalent plastic strain and elastic strain energy of element 5 increases noticeably, meaning element 5 is the main deformation position. It is worth noting that the equivalent plastic strain of element 4 does not increase noticeably, but the elastic strain energy does noticeably increase. The elastic strain energy value of element 4 gradually increases to the maximum. In this case, springback will increase with increasing pressure. This corresponds to Case 2 in Section 3.1, that is, the springback value is positively correlated with the degree of deformation when the pressure is in the range of 80–100 MPa. In the “CD” segment, all elements of the upper fillet have almost completed deformation, so the equivalent plastic strain of all elements does not change much. But the elastic strain energy of element 4 still increases with increasing degree of deformation, which indicates that the deformation of element 4 is elastic in the “CD” segment. This is inconsistent with the negative correlation between springback value and deformation degree in Case 3, so it is necessary to analyze the influence of other positions on springback.

**Figure 16:**
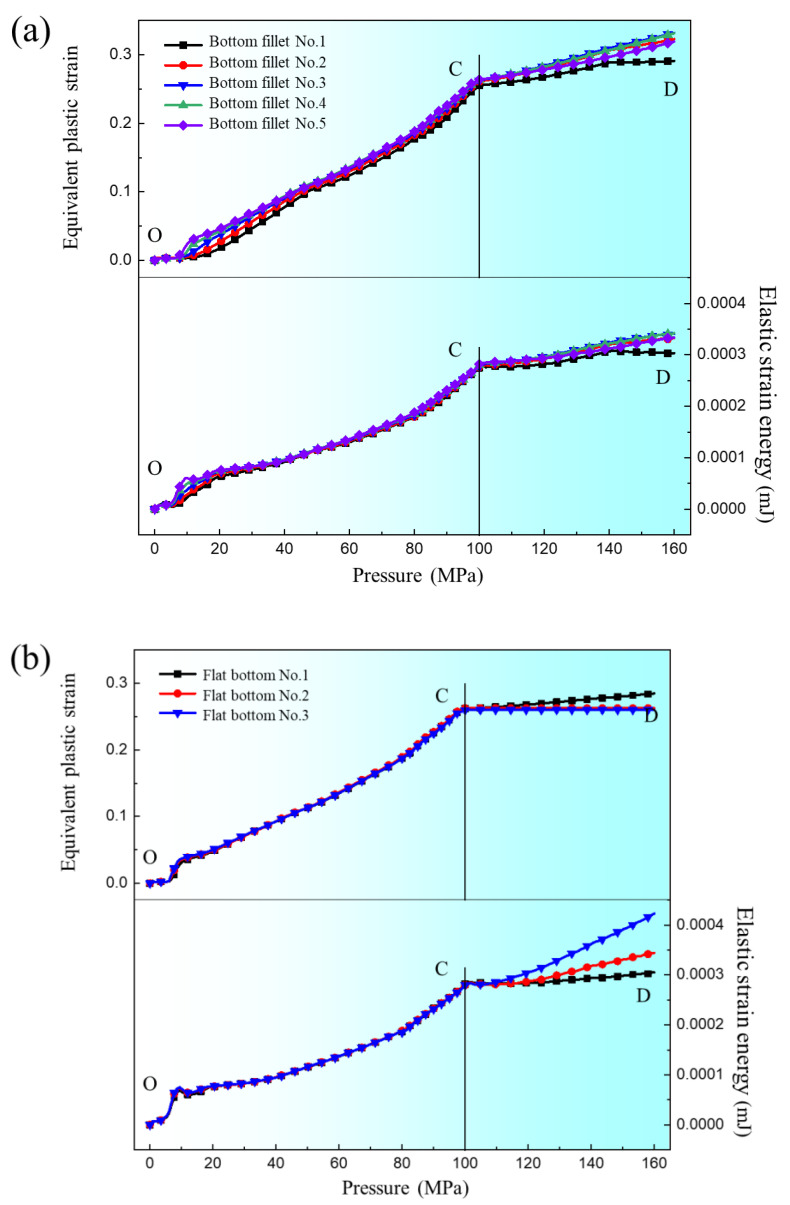
Equivalent plastic strain and elastic strain energy change with internal pressure at different points: (**a**) bottom fillet; (**b**) flat bottom.

Figure 16 is the equivalent plastic strain and elastic strain energy results of the five elements of the bottom fillet and the three elements at the flat bottom of the channel. As can be seen from Figure 16a, the equivalent plastic strain and elastic strain energy of the five elements in the bottom fillet are similar, and all increase with increasing pressure. An inflection point exists at position “C”. In Figure 16b, the results of the three flat bottom elements are similar to those at the bottom fillet, except that the elastic strain energy of flat bottom element 3 still increases significantly in the “CD” segment. In Case 3 in Section 3.1, when the internal pressure exceeds 100 MPa, the deformation of the upper fillet is nearly complete. The flat bottom of the channel (flat bottom element 1) is attached to the die and the deformation is almost complete, hence the equivalent plastic strain is basically unchanged. At this point, the bottom fillet continues to deform, and the springback is mainly caused by upper fillet element 4 and flat bottom element 3. As the degree of deformation increases, the equivalent plastic strain continues to increase while the increase in elastic strain energy is not significant, which plays a major role in suppressing springback, resulting in a negative correlation between the springback value and the degree of deformation.

**Figure 17:**
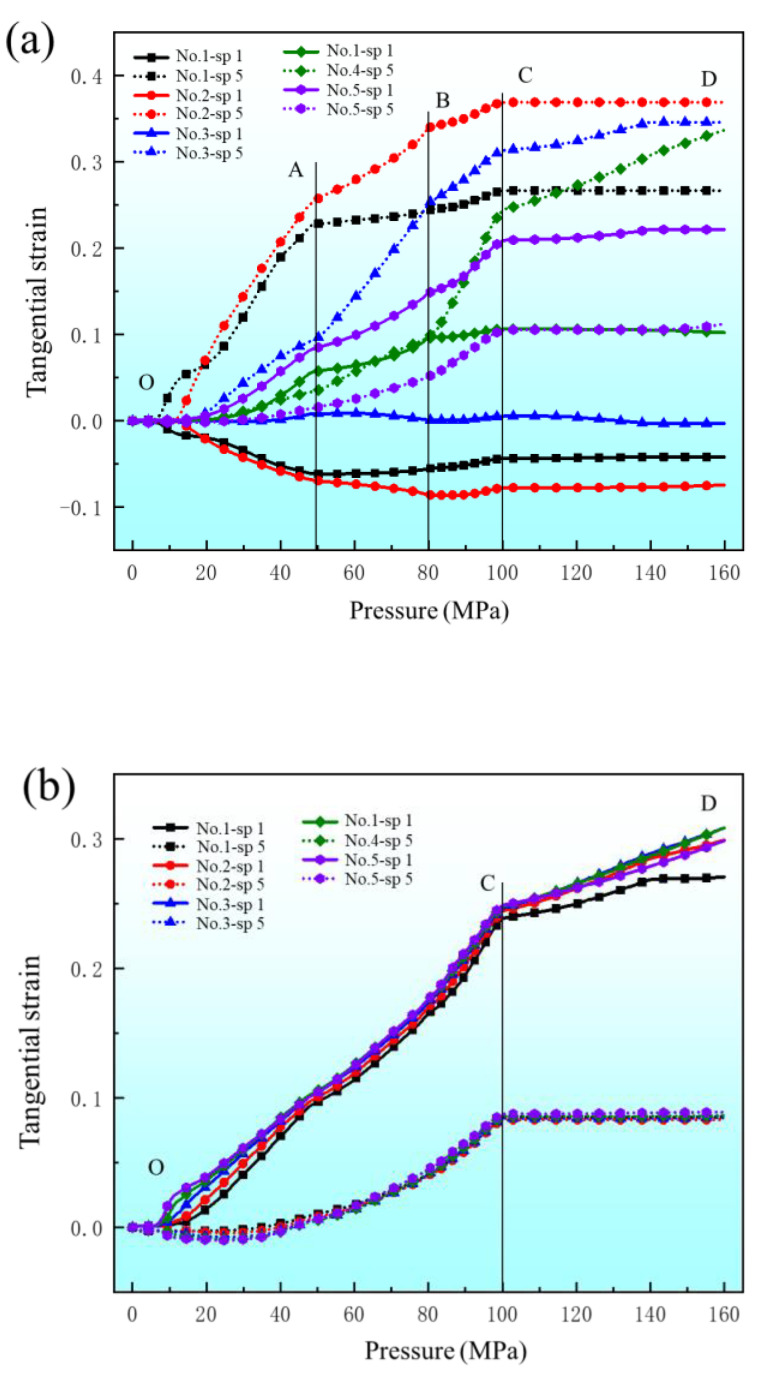

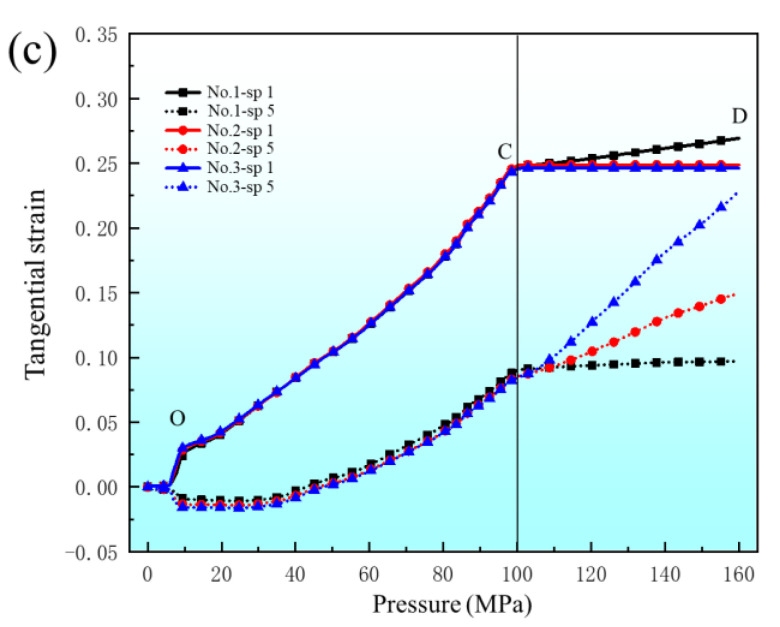
The tangential strain changes with internal pressure at different points: (**a**) upper fillet; (**b**) bottom fillet; (**c**) flat bottom.

Figure 17 shows the change of tangential strain at different positions with increasing degrees of deformation (increasing pressure), where the tangential strain of the bottom surface in the direction of plate thickness is SP1 and the tangential strain of the top surface in the direction of plate thickness is SP5. As can be seen in Figure 17a, upper fillet elements 1 and 2 show typical bending characteristics, that is, SP1 is tangential compression strain and SP5 is tangential tensile strain. The SP1 and SP5 of upper fillet elements 4 and 5 are tangential tensile strain, indicating that elements 4 and 5 are under tension across the entire cross-section. The SP5 tangential strain of upper fillet element 4 increases sharply in the “BC” segment, while SP1 tangential strain is almost unchanged. For upper fillet element 4, in the “OB” segment, the tangential strain values of SP1 and SP5 are basically equal and in a state of uniform tension, while in the “BC” segment, the bottom of element 4 just contacts the die. So, the increasing degree of deformation is due to the friction generated by the contact between the bottom and the die. The SP1 tangential strain does not increase significantly during the tensile deformation, while the top is not constrained by the die, and the tensile strain is large. The deformation of element 4 is uneven. In the direction of thickness, the tangential tensile deformation increases from the bottom to the top, while the tangential strain at the bottom is small. According to the elastic-plastic mechanics of the metal material, the elastic strain part will first increase and then remain unchanged in the direction of thickness, resulting in an increase in the elastic region in general, especially in the bottom region.

As shown in Figure 17b,c, the variation law of tangential strain and equivalent plastic strain at the bottom fillet and the flat bottom of the channel are similar. The SP1 tangential strain of all elements at the bottom fillet and the flat bottom of the channel is greater than the SP5 tangential strain.

**Figure 18:**
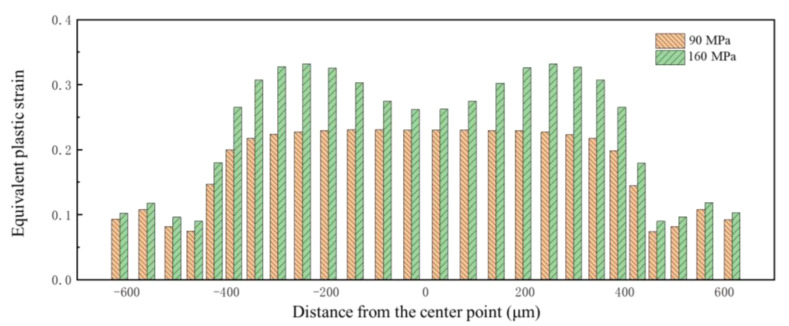
Distribution of equivalent plastic strain along the channel under different pressures.

Pressures of 90 MPa and 160 MPa were selected as the research objects; these pressures lead to the bottom of the bipolar plate not attaching to the die, and attaching to the die, respectively. The equivalent plastic strain at different points along a cross-section of the channel is shown in Figure 18. It can be seen that there is a significant difference at the bottom fillet. The distribution of equivalent plastic strain corresponds to the results of springback shown in Figure 13. When the flat bottom of the channel is attached to the die, deformation mainly affects the upper fillet, and the point with the greatest springback is also the upper fillet.

**Figure 19:**
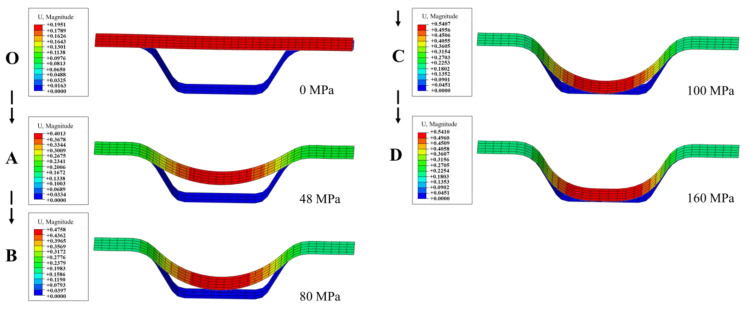
Contour changes over pressure (The arrows in the figure indicate changes in the forming process).

Figure 19 shows the change of channel profile over pressure. In the “OA” segment, the upper elements of the upper fillet are in contact with the die. In the “AB” segment, the overall equivalent plastic strain increases while the proportion of elastic strain decreases. In the “BC” segment, the deformation of upper fillet element 4 is uniform with more elastic regions. In the “CD” segment, the deformation of the upper fillet and the bottom of the channel is basically completed, and the proportion of elastic strain on the bottom fillet becomes lower and lower as deformation continues.

### 3.3. Effect of Grain Size on Forming and Springback

**Figure 20:**
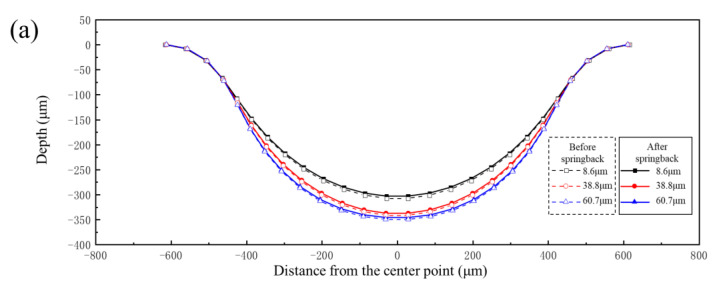

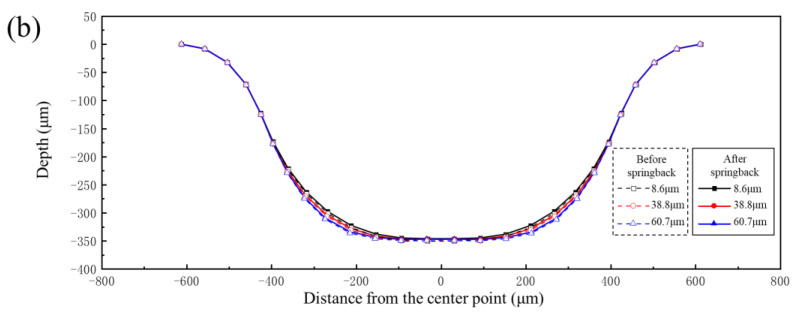
The forming profile and springback profile of bipolar plates with different grain sizes: (**a**) 90 MPa; (**b**) 160 MPa.

The mechanical characteristic parameters of different grain sizes shown in Figure 3 were input into the finite element model. Pressures of 90 MPa and 160 MPa were also selected as the research objects. The forming profiles and springback profiles of channels of bipolar plates with different grain sizes are shown in Figure 20. The forming depth of the bipolar plates was 303 μm, 337 μm and 347 μm for grain sizes of 8.6 μm, 38.8 μm and 60.7 μm under pressure of 90 MPa, respectively. The flat bottom of the channel for all three grain sizes was attached to the die under pressure of 160 MPa, and the larger the grain size, the better the degree of attachment to the die. Springback is closely related to the elastic modulus; as shown in Figure 3, the elastic modulus of different grain size materials is the same, but the strength is obviously different. Smaller grain sizes result in greater resistance to deformation, which is not conducive to plastic deformation. On the other hand, as grain size increases, the number of grains per unit thickness decreases, leading to fewer grain boundaries per unit area. This reduction in grain boundary strengthening makes the material more prone to plastic deformation. Therefore, under the same loading conditions, sheets with larger grains exhibit better forming performance.

**Figure 21:**
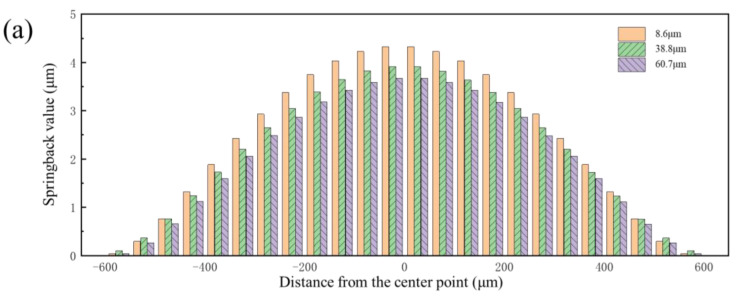

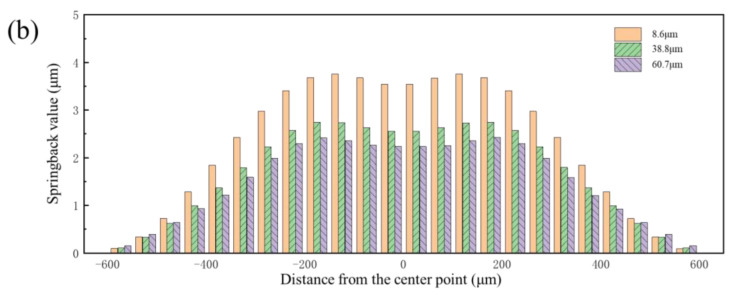
Springback value distribution along the cross-section of channels with different grain sizes: (**a**) 90 MPa; (**b**) 160 MPa.

**Figure 22:**
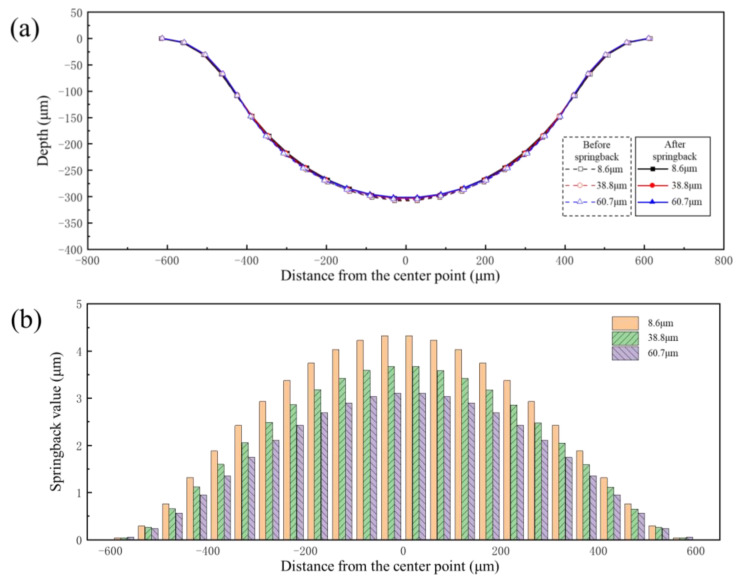
The results of different grain sizes under the same degree of deformation: (**a**) profile distribution; (**b**) springback distribution.

Figure 21 shows the springback value distribution along the cross-section of the channel with different grain sizes under the same pressure. It can be seen from the results that the springback value decreases with increasing grain size under two levels of pressure. Due to the different degrees of deformation of varying grain sizes under the same pressure (Figure 20), it is impossible to clarify the influence of grain size on springback. Therefore, the springback values of three grain sizes under the same degree of deformation were compared. Figure 22a shows the forming profiles and springback profiles of three grain sizes at the same degree of deformation (the channel depth is 303 μm). Figure 22b shows the springback value distribution along the cross-section of the channel with different grain sizes under same degree of deformation. The results indicate that springback decreases with increasing grain size after forming. The reason for this phenomenon is that sheets with larger grain sizes have fewer grain boundaries and dislocations, which means reduced stress concentration within the grains and thus a decreased tendency for springback. Therefore, larger grain sizes in the sheet are more beneficial in reducing springback after forming.

## 4. Application of Deformation Behavior and Springback Law

**Figure 23:**
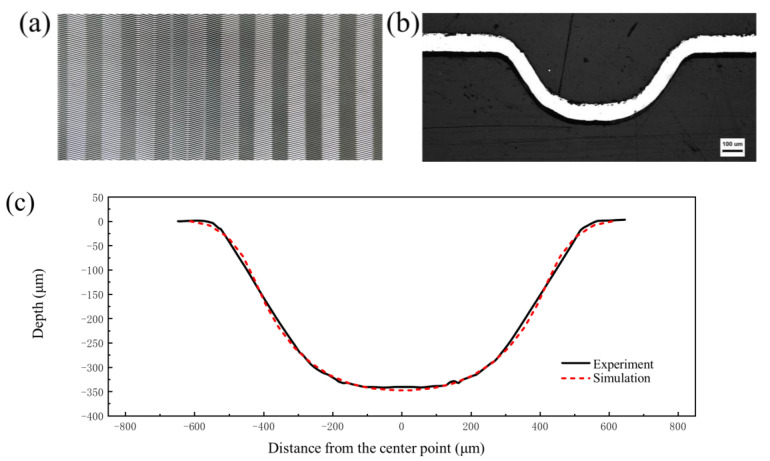
(**a**) Photograph of the formed product; (**b**) metallographic photo of a single flow channel; (**c**) comparison of simulation and experiment.

In the hydroforming process of bipolar plates, increasing the pressure is necessary to maximize the depth of the forming channels. However, increasing the internal pressure requires higher equipment tonnage and higher sealing requirements. Based on the study of forming behavior and springback law in this paper, the following parameters were selected to achieve the goals of maximizing channel depth, minimizing springback value, and requiring lower pressure: a grain size of 60.7 μm (achieved by holding at 1050 °C for one hour) and an internal pressure of 100 MPa. Figure 23a shows a photo of the shaped bipolar plate. Figure 23b shows a metallographic photo of a single flow channel. Figure 23c is a comparison between the profile of the formed channel (measured by white light interference) and the finite element simulation. It can be seen from the results that the bottom of the channel has been attached to the die and a flat bottom appears, and the forming quality is good, which is of high significance for the production of bipolar plate hydroforming.

## 5. Conclusions

In order to realize the precision forming of an ultra-thin metal bipolar plate with micro-size channels, the influence of degree of deformation and grain size on the forming and springback behavior of the bipolar plate in the hydroforming process was studied. The springback behavior of the bipolar plate after forming was explained by analyzing the equivalent plastic strain and elastic strain energy of the bipolar plate at different stages of deformation. The main conclusions are as follows:

(1) With increasing forming pressure, the degree of deformation increases, and the springback value initially decreases, followed by an increase, but then again decreases. In the early segment of deformation (pressure < 80 MPa), the plastic deformation of the bipolar plate increases while the proportion of elastic strain decreases; in the middle segment of deformation (80 MPa < pressure < 100 MPa), the deformation of the lower element of the upper fillet is uniform with more elastic regions; in the later segment of deformation (pressure > 100 MPa), the deformation of the upper fillet and the bottom of the channel is basically completed, and the elastic strain proportion of the bottom fillet becomes lower and lower as deformation continues.
(2) The location of maximum springback is different when the bottom of the bipolar plate is attached to the die or not attached to the die. When the deformation of the bipolar plate does not reach the extent of attaching to the bottom of the die, the springback value gradually decreases from the center of the bottom towards the sides; when the deformation of the bipolar plate is enough for the bottom of the bipolar plate to attach to the die, the springback value distribution along the cross-section becomes “M”-shaped.
(3) Under the same loading conditions, sheets with larger grain sizes exhibit better forming performance. The forming depth of the bipolar plates was 303 μm, 337 μm and 347 μm for grain sizes of 8.6 μm, 38.8 μm and 60.7 μm, respectively, under pressures of 90 MPa. Sheets with smaller grain sizes exhibit greater resistance to deformation and are less conducive to plastic deformation.
(4) Under the same degree of deformation, the springback value decreases with increasing grain size. The reason for this phenomenon is that sheets with larger grain sizes have fewer grain boundaries and dislocations, which means reduced stress concentration within the grains and thus a decreased tendency for springback.
(5) The grain size has great influence on the forming quality of the bipolar plate. In future work, the springback behavior, thickness distribution, surface roughness and corrosion resistance of plates with different grain sizes should be the focus of research, and it is also necessary to study the deformation behavior of different materials.

## Figures and Tables

**Table 1:** Values of channel parameters.

Parameters	$L_{1}$	$L_{2}$	$L_{3}$	α	h	r	R	$L_{4}$
Values	8.99	0.49	0.31	60	0.35	0.1	0.2	1.55

**Table 2:** 316L stainless steel chemical composition (wt.%).

C	Cr	Ni	Mn	Si	Mo	P	S
0.026	16.68	10.10	1.32	0.50	2.02	0.028	0.0017

**Table 3:** The properties of 316L stainless steel.

Grain Size (μm)	E (GPa)	v	$\sigma_{y}$ (MPa)	K (MPa)	n	$\varepsilon_{0}$
8.6	196	0.3	323	1776	0.62	0.06
38.8	196	0.3	247	1661	0.67	0.06
60.7	196	0.3	213	1487	0.70	0.06

## Data Availability

The original contributions presented in the study are included in the article.
